# The cost of non-coordination in urban on-demand mobility

**DOI:** 10.1038/s41598-022-08427-2

**Published:** 2022-03-18

**Authors:** Dániel Kondor, Iva Bojic, Giovanni Resta, Fábio Duarte, Paolo Santi, Carlo Ratti

**Affiliations:** 1 Singapore-MIT Alliance for Research and Technology, 1 Create Way, Singapore, 138602 Singapore; 2 Istituto di Informatica e Telematica del CNR, Via Giuseppe Moruzzi, 1, 56127 Pisa, Italy; 3 Senseable City Laboratory, MIT, 77 Massachusetts Ave, Cambridge, MA 02139 USA; 4 Pontificia Universidade Catolica do Parana, R Imaculada Conceicao 1155, Curitiba, PR 80215-901 Brazil

**Keywords:** Applied mathematics, Computer science

## Abstract

Over the last 10 years, ride-hailing companies (such as Uber and Grab) have proliferated in cities around the world. While generally beneficial from an economic viewpoint, having a plurality of operators that serve a given demand for point-to-point trips might induce traffic inefficiencies due to the lack of coordination between operators when serving trips. In fact, the efficiency of vehicle fleet management depends, among other things, density of the demand in the city, and in this sense having multiple operators in the market can be seen as a disadvantage. There is thus a tension between having a plurality of operators in the market, and the overall traffic efficiency. To this date, there is no systematic analysis of this trade-off, which is fundamental to design the best future urban mobility landscape. In this paper, we present the first systematic, data-driven characterization of the cost of non-coordination in urban on-demand mobility markets by proposing a simple, yet realistic, model. This model uses trip density and average traffic speed in a city as its input, and provides an accurate estimate of the additional number of vehicles that should circulate due to the lack of coordination between operators—the cost of non-coordination. We plot such cost across different cities—Singapore, New York (limited to the borough of Manhattan in this work), San Francisco, Vienna and Curitiba—and show that due to non-coordination, each additional operator in the market can increase the total number of circulating vehicles by up to 67%. Our findings could support city policy makers to make data supported decisions when regulating urban on-demand mobility markets in their cities. At the same time, our results outline the need of a more proactive government participation and the need for new, innovative solutions that would enable a better coordination of on-demand mobility operators.

## Introduction

In the last 10 years, urban mobility has gone through disruptive changes, greatly caused by the appearance and success of Transportation Network Companies (TNCs)^1,2^. In less than 2 years since Uber’s launch in 2009, there were as many as 638 ride-hailing companies in North America alone^3^. While providing a convenient travel option for many passengers, TNCs have been criticized for exacerbating traffic problems in cities globally^4,5^. Cities are thus increasingly looking at policy and regulatory tools to mitigate negative externalities while fostering affordable and reliable on-demand mobility options for residents^6–9^.

There are multiple inefficiencies in current taxi and TNC operations that limit service quality while adding to congestion. Recent research has shown that the number of circulating taxis in Manhattan could be reduced by as much as 40% while serving the same demand if fleet operations were centrally controlled and optimized^10^. Beside ineffective operational optimization, excess drivers and vehicles are on the roads also due to market reasons^11,12^. While the operational strategies of TNCs are typically not publicly available, it is generally understood that TNCs do not aim to limit the number of drivers nor have measures in place to limit empty travel; fleet rebalancing is only performed by using surge pricing as an incentive for drivers to move to areas with high demand. Recognizing this, recently the city of New York initiated regulation for TNCs that considers fleet-wide efficiency as an important measure^6^, while several other cities are aiming to implement regulations that limit the number of vehicles on the road^7,8^.

Beside these factors, an additional cause of inefficiencies is segmentation of demand among multiple competing taxi and TNC operators (we refer to this as a segmented market as opposed to a monopolistic market that would exist if only one operator was present). In an operational model with central dispatching (as is the case of TNCs and increasingly with taxis), having access to a larger share of the demand can offer additional opportunities for optimizing the fleet and reduce empty travel kilometers, effectively exploiting economies of scale. While there is a body of literature that studies how to assign a vehicle to a certain passenger in an optimized way, revealing significant possible benefits compared to current taxi operations (e.g.^13–16^), these works typically assume a single operator in the market and they thus overlook the effects of segmented demand. Vazifeh et al. showed that in the case of the New York taxi market, segmenting demand across multiple non-coordinated operators could imply an increase in total fleet size of up to 10% compared to the monopolistic case^10^. However, these results are not easily generalizable to different cities that are expected to have different constraints on operator efficiency.

Besides existing literature focusing on optimizing fleet size for either single (e.g.^10,17^) or shared rides (e.g.^18,19^) while overlooking the effects of demand segmentation, there is also ample research looking at market segmentation in TNC field from an economical point of view^12,20,21^. For example, in^22^ the authors examined competition amongst different TNC platforms and showed that when passengers have installed and used different apps (i.e. multi-homing), driver idle time falls compared to the case of a monopolistic market. On the other hand, it was shown that multi-homing is socially superior to single-homing only when platforms are symmetric^23^. Recently, Séjourné et al. showed that competition among multiple operators directly translates to an increase in empty miles traveled, with a strong dependence on the structure of demand^24^. Even more, the rise of monopolistic TNCs in Asia (e.g. Grab and DiDi) also showed some greater social impact, despite of changes in pricing, insurance and benefits for drivers^25^.

We would like to make here a distinction between *market segmentation*, i.e., the coexistence of multiple operators that serve the same demand pool for urban mobility, and *demand segmentation*, which occurs when operators serve the demand pool independently of each other in a non-coordinated, competitive model. This distinction is important because the economic benefits for passengers are generated by *market* segmentation, while the inefficiencies in fleet management are induced by *demand* segmentation. While market and demand segmentation co-exist in current urban mobility markets, a different model in which multiple operators co-exist in a urban market while all drawing from a shared, centrally coordinated demand pool could be promoted. One of the main objectives of this paper is to provide a quantitative estimate of the benefits that this model would provide to the entire urban mobility system.

Our paper fills the gap between the two aforementioned lines of work. We present a systematic characterization of the cost of demand segmentation in on-demand mobility. More specifically, we measured the additional number of cars needed to serve a given demand pool when there is competition between multiple non-coordinated operators, compared to the case when the demand pool is served in a coordinated way. This way, our work has a direct relevance to cities facing externalities such as increased congestion due to additional vehicles on the road^20^. Our aims are similar to Ref.^24^, but instead of utilizing a theoretical solution based on aggregate travel demand, we base our main results on simulations of taxi operations serving the actual sequence of trips. Our work is also complementary to Ref.^24^ in that we are focusing on fixed costs (i.e. fleet size) instead of variable externalities represented by travel distance. By using a data-driven approach utilizing taxi trip data from five cities, we are able to estimate the increase of the total number of vehicles circulating in the city due to non-coordination among multiple operators serving the given demand pool. Moreover, we developed a simple model for calculating the cost of demand segmentation, i.e., the *cost of non-coordination*, in a particular city using only its average trip density and average traffic speed.

## Results

In this paper, we aim to compare the fleet size requirements of on-demand mobility operators with different market shares. In our simulation, we used samples of the real-world demand data collected for 21 days in five cities across the world: Singapore, New York (limited to the borough of Manhattan in this work), San Francisco, Vienna and Curitiba. These represent taxi markets with different local properties, such as city size, traffic speed, density of trips (see Table S1 in the Supplementary Information (SI) for more information). In our simulation, we implemented a dispatching strategy with random cruising for the relocation of vehicles. Finally, while calculating the minimum fleet size, we require that 95% of passengers are served within maximum of 5 min of waiting time.

### Definitions and assumptions

We denote by *p* the share of the total demand pool served by a specific operator; $p = 1$ corresponds to monopoly, while $p < 1$ corresponds to an operator that has access to a subset of the market. In the current work, such on-demand mobility operators include both traditional taxis and TNCs. We assume such operators to use a form of central dispatching, as is standard for TNCs and increasingly common for taxis. One operator corresponds to an entity with such a central dispatching system; whether such company owns any vehicles or only connects passengers to drivers is irrelevant for the sake of the following analysis. Also, when estimating fleet size, and, hence, the cost of non-coordination, we make the assumption that, when multiple operators are present in a market, their centralized dispatching systems operate independently for the sole purpose of serving the share of demand of each single operator. I.e., there is no coordination between operators in order to serve the overall demand pool. We assume a fixed demand for on-demand mobility, and passengers who only use one out of multiple potential operators. This is a simplification from the real-life case where passengers can install multiple ride-hailing apps; however, real-time comparison of mobility offers by competing operators is burdensome as it requires opening and switching between multiple apps with changing information.

We then denote by $N_V (p)$ the fleet size (i.e. number of vehicles) required for an operator with demand share *p* to provide adequate service to its passengers. In the following, we focus on relative differences between operators with different demand shares as a measure of inefficiency due to demand fragmentation, measured by the *fleet size factor*, defined as the following:

1 $$\begin{aligned} R (p) = \frac{N_V (p)}{p N_V (1)} - 1 \end{aligned}$$

Here, *R*(*p*) quantifies the relative increase in fleet size due to inefficiencies, which we take as a cost of non-coordination. In an ideal case, where $N_V$ is exactly proportional to *p*, we would have $R(p) = 0$ for any value of *p*. On the other hand, if having access to a larger share of the market allows additional optimization opportunities, *R*(*p*) will be a monotonically decreasing function of *p*. We note that beside an increase in fleet size, additional externalities that could be present include increased emissions and congestion due to additional travel of the fleet; these will depend on the cruising and relocation strategy of the operators^15,16,24^ and are thus out of scope of the current work. Regardless of cruising strategy used, we expect that increases in fleet size will translate into additional travel and use of valuable real estate in city centers in the form of waiting and parking spaces^17^. Furthermore, beside capital costs for operators or drivers, a larger fleet translates into additional externalities in terms of the energy and resources needed for the manufacturing, maintenance and disposal of the vehicles.

In a generic case, we can assume that the fleet size required to adequately serve a set of trips that happen in a day ($N_T$) follows a linear relationship as:

2 $$\begin{aligned} N_V = A N_T + B \end{aligned}$$

where *A* and *B* are parameters that can be different for each city, based on local properties (such as shown in Tab. S1 in the SI). For simplicity, we assume a constant fleet size during the day, but our analysis could be extended for a case where we consider the maximum of a varying fleet size. The first term in Eq. (2) can be seen as the number of busy vehicles, i.e. either serving a trip, or on their way to pick up a passenger. The second term represents the number of drivers who are idle at any time. In an ideal case, no drivers would be idle, as whenever a driver finishes a trip, they would be assigned to an upcoming trip. However, this would only be possible if the sequence of trips is perfectly aligned to allow such connections in an ideal fashion, e.g. if for each passenger dropped off, there is a next passenger at a nearby location who wants to start a new trip. While predictive fleet rebalancing and optimized assignment of drivers to requests is expected to help mitigate such inefficiencies^10,13–16^, an operator aiming to provide *reliable* service still needs to ensure that at any point in time, there are enough idle drivers available in the city to serve *any* possible trip request in a short time, even if it is not expected based on demand predictions. This means that in any real-world scenario, a value of $B > 0$ will be needed unless trip origins and destinations are highly balanced.

Based on the above considerations, the fleet-size factor can then be expressed as the following:

3 $$\begin{aligned} R(p) = D \, \left( \frac{1}{p} - 1 \right) \end{aligned}$$

where the constant *D* is defined as:

4 $$\begin{aligned} D \equiv \frac{b}{A n_T + b} \end{aligned}$$

Here, for a city of area $|{\mathscr{C}}|$, we define $n_T \equiv N_T / |{\mathscr{C}} |$ as the trip demand density (i.e. number of trips per square kilometre per day) and $b \equiv B / |{\mathscr{C}} |$ as the density of idle drivers. This way, the explicit dependence on city size is removed from *D*. In the rest of paper, we will focus on *D* as a city-specific factor, rather than on the individual constants *A* and *b*. In addition to the density of trips in the city, the constant *D* will typically depend on additional relevant city parameters such as average trip density, traffic speed or trip length, or on the actual distribution of demand in space.

While the fleet size factor for each operator is highly dependent on the demand share, the increase of the *combined* city-wide fleet of vehicles only depends on the number of operators added. If we consider that the on-demand mobility market in a city is served by *m* operators, each with a demand share $p_i$ ($i = 1,2\ldots m, \sum _i p_i = 1$), then we can calculate the combined fleet size as:

5 $$\begin{aligned} N_V^{(m)} = \sum _{i = 1}^m N_V (p_i) = A N_T + m B \end{aligned}$$

The relative increase compared to the case of one operator with monopoly is then:

6 $$\begin{aligned} \frac{N_V^{(m)}}{N_V} - 1 = (m - 1) D \end{aligned}$$

In this way, *D* gives the relative increase in the city-wide fleet size for each additional operator who enters the market, regardless of the distribution of individual operators’ demand share.

### Empirically calculating fleet size factors

**Figure 1:**
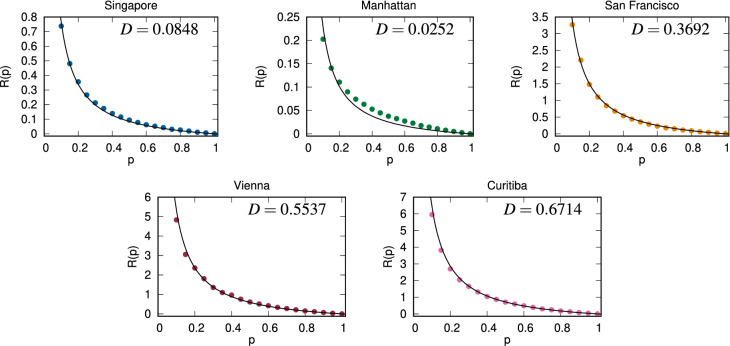
Fleet size factors for five cities determined via simulations as a function of demand segmentation, i.e. the share of trips an operator is serving. The lines are best fit curves of the relationship $R(p) = D (1/p - 1)$; fitted values of *D* are shown in the individual panels.

We perform simulations in five cities based on real-world taxi data to gain empirical estimates on the dependence of the fleet size factor *R*(*p*) on the presumed demand share of on-demand mobility operators. An overview of the cities and the datasets is presented as Table S1 in the SI. We use taxi data as a proxy for mobility demand in cities; we expect our results to be generalizable for a case where TNC trip data is available as well.

In these simulations, we use a First-Come-First-Served (FCFS) model of dispatching along with a simple model of idle vehicle relocation based on aggregate demand, as described in more detail in the Materials and Methods section and in the SI. For each city, we arrive at minimum fleet size requirements by running Algorithm S1. We repeated this procedure for subsets of the trips, selected by simple random sampling, varying the demand share *p* between 10 and 100% in 5% increments. This way, we gained a set of $(N_T, N_V)$ pairs that we display in Fig. S1 in the SI to confirm the linear relationship of Eq. (2). We note that these results were obtained under the assumption that all vehicles are active during the whole day; a natural extension of our work would optimize for the instantaneous fleet size, i.e. keeping the number of active vehicles minimal at any point in time during the day. While this is out of scope of the current work, we note that we can expect that daily maximum fleet sizes would be at least as large as the $N_T$ values calculated in our current simulations.

Then, for each city and each value of *p*, we calculate the average of corresponding *R*(*p*) values among all days as our estimate of the effect of demand segmentation and display these results in Fig. 1. A point in Fig. 1 means that when an operator is serving only a smaller share of trips, it would need a higher relative fleet size compared to what we expect by a simple rescaling of the fleet size for $p = 1$. Finally, the black curve in each sub-figure is the form described by Eq. (3) using the best-fit *D* value.

From Fig. 1, we can conclude that the calculated values in our simulations are indeed following the shape described by the equation $R(p) = D (1/p - 1)$. However, the values on the *y*-axes are different, which means that different cities are not equally susceptible to demand segmentation. For example, in the case of Manhattan, each additional operator will contribute to an increase of the city-wide fleet size by 2.52% in accordance with Eq. (S3) in the SI. In Singapore, this increase is 8.48%, while in Curitiba, it climbs to 67.14%. In accordance with Eq. (3), the cost of non-coordination is borne by operators unevenly: in Singapore, in a hypothetical scenario with three operators with demand shares of 50%, 35% and 15%, additional fleet needed by the operators compared to the monopolistic case is 8.48%, 15.75% and 48.05%, compared to a city-wide combined increase of 16.96%. We can see that the cost of non-coordination is mainly borne by the smallest operator. This suggests that larger players have a significant market advantage, and competing against them is especially challenging, as such inefficiencies will translate to lower earnings for the drivers and the platform.

**Figure 2:**
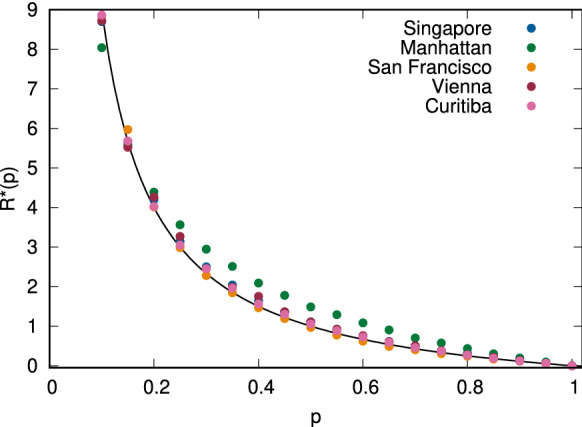
Rescaled average fleet size factor for all cities. All results have been scaled with the best fit *D* values identified based on the results displayed in Fig. 1.

To compare across different cities, we have rescaled the *R*(*p*) values from each of the panels of Fig. 1 by dividing them with the corresponding *D* value, getting the generic curve $R^*(p) = (1/p - 1)$. We display the resulting curves in Fig. 2. We observe that most cities follow the same curve well, with Manhattan showing some deviations. Nevertheless, the function form for *R*(*p*) described in Eq. (3), derived from a set of simple assumptions, works very well for a wide range of cities and demand segmentation impacts.

### Modelling the cost of non-coordination

In the previous section, we empirically showed that the dependence of the fleet size factor *R*(*p*) can be well modeled with the universal form $R(p) = D (1/p - 1)$. Moreover, from Eq. (4) it is evident that the constant *D* is city dependent and is monotonically decreasing with the average trip density $n_T$. In order to determinate how the constant *D* depends on other city parameters such as average trip length, traffic speed or trip duration, for each city we generate a set of synthetic datasets according to the random model developed in^19,26^. For each city, we synthetize new trips based on the distribution of trips in the original datasets. In this way, we could perform a more in-depth comparison of market segmentation effects across different cities, by allowing us to use synthetic datasets that have the same density of trips among all cities.

More concretely, we initially calculated trip densities for our five cities as recorded in their corresponding original datasets (as shown in Table S1). Then, for each city, we synthesized nine additional datasets by generating the necessary number of trips needed to achieve a certain trip density. Five of those densities were based on the densities recorded in the initial datasets for five cities, while other five densities were selected to better cover the range among these values. We were thus able to better estimate the dependence between the constant *D* and the other city parameters from Table S1 by keeping the trip density constant across all cities.

In the SI, (1) Fig. S2 shows how the constant *D* depends on the average trip distances; (2) Fig. S3 shows how the constant *D* depends on the average trip duration; (3) Fig. S4 shows how *D* depends on the average traffic speed (as computed from the initial trip datasets for each dataset). From these, we can conclude that for a constant trip density, *D* decreases monotonically with increasing traffic speed, while dependence on the other variables is not significant. We note that the aforementioned parameters are not fully independent; e.g. the average trip duration will obviously depend on trip distances and traffic speed. However, based on the results presented in SI Figs. S2–S4, we chose to build a simple empirical model that only depends on the average trip density and traffic speed.

**Figure 3:**
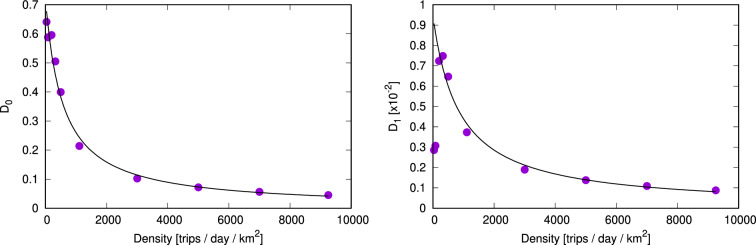
Coefficients $D_0$ and $D_1$ (left and right panel respectively) from the fitted lines in Fig. S4 in the SI as a function of trip density.

Results from Fig. S4 in the SI show that the constant *D* follows an approximate linear trend as a function of an average travel speed. The observed correlation is strong for higher densities (i.e. more than 200 trips per day per $\hbox {km}^2$), and is a bit weaker for lower densities, which are for example observed in Vienna and Curitiba. Essentially, we have the following, empirical relationship: $D = D_0 - D_1 v$, where the $D_0$ and $D_1$ values depend on the average density of trips in the city. As the next step, we then relate the $D_0$ and $D_1$ coefficients in the best linear fit to the average density of trips and display their values in Fig. 3. What we find is that both $D_0$ and $D_1$ coefficients can be well approximated with the following functional form consistent with Eq. (4):

7 $$\begin{aligned} D_i = \frac{E_i}{n_T + F_i} \end{aligned}$$

Putting this together, we finally have the following empirical model of *D* as a function of trip density and travel speed:

8 $$\begin{aligned} D = \frac{E_0}{n_T + F_0} + \frac{E_1 v}{n_T + F_1} \end{aligned}$$

with the values of the coefficients being $E_0 = 407\,\mathrm {day}^{-1} \mathrm {km}^{-2}$, $F_0 = 564\,\mathrm {day}^{-1} \mathrm {km}^{-2}$, $E_1 = 0.36\, \mathrm {km}^{-3}$, $F_1 = 819\,\mathrm {day}^{-1} \mathrm {km}^{-2}$.

### Verification of our model

**Figure 4:**
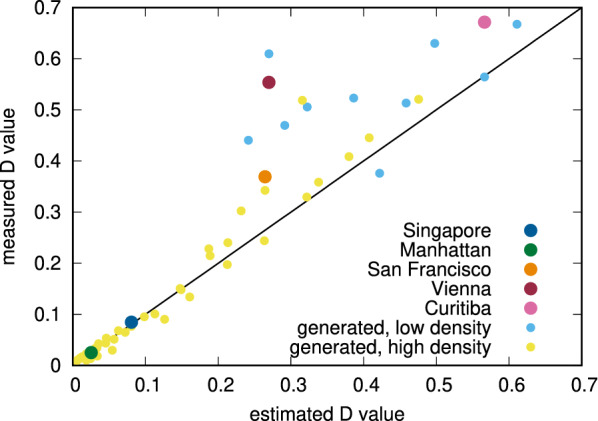
*D* coefficients according to our simulation results and the empirical model from Eq. (8).

We can then use Eq. (8) to predict the expected value of *D* in any city, based on the density of trips and average traffic speed. Fig. 4 present the results for both original datasets, as well as for all generated ones. We divided average trip densities $n_T$ into two groups, distinguishing between cases of high and low densities. Using 200 trips per day per $\hbox {km}^2$ as the threshold, from the original datasets, Singapore, Manhattan and San Francisco qualify as high density cases and Vienna and Curitiba as low density ones. We note that as evident in Fig. 3, the model for the $D_1$ coefficient loses its validity at such low densities. Consequently, we were able to predict the cost of non-coordination with $R^2 = 0.83$ across the whole dataset and with $R^2 = 0.92$ if we restrict the results to only high-density cases. We note that the model performs remarkably well among a large range of cases for high densities from 200 to 10,000 trips per day per $\mathrm {km}^2$.

Other important parameters, such as the typical length of trips could be incorporated to try to explain deviations, especially for lower density cases. Nevertheless, since using our datasets we are not able to independently vary trip length, distance and traffic speed, we chose to present a simpler model that already shows remarkably good predictive power for the effect of demand segmentation in cities with typical trip densities. We also note that worse performance for low trip densities could also be the result of limitations in our simulation methodology. Essentially, our methodology can achieve adequate performance by effectively ignoring parts of the city which have very low demand in terms of trips (i.e. drivers are never relocated to these neighborhoods). This means that our simple assumption for a constant *B* in Eq. (2) will not hold anymore in practice. On the other hand, an operator aiming to gain reputation as a provider of a *reliable* service might still need to consider having a sufficient stand-by fleet to serve neighborhoods with low demand, meaning that the real-world fleet size demand might be more similar to the relationship described by Eq. (2) instead of the results of simulations.

### Oracle model

While the above results present a good estimate of the effect of demand segmentation under the realistic conditions, there are many potential improvements that are possible to achieve based on better prediction of demand. For example, if advance knowledge of all trips in a day is available, then this could be used to construct a *shareability network* of trips, and then an ideal dispatching strategy that contains trip chains served by each vehicle in the fleet could be selected. The oracle model can be interpreted as a best case scenario of demand prediction, where the predicted demand always match future trip requests. To evaluate the extent to which such predictions might help operators, we calculated theoretical minimum fleet sizes required to serve all trips in the taxi datasets in the five cities using the methodology of Vazifeh et al.^10^. The result of this process is a fleet size that can serve all trips without any delay. For each taxi dataset, we repeated the subsampling process for presumed demand shares between 10 and 100%, gaining an empirical estimate of fleet size factors, denoted $R^{(O)}(p)$ in this case. In Fig. S5 in the SI, we display these results along with *R*(*p*) calculated in the FCFS model for the five cities.

Fleet size factors in the case of the Oracle model are significantly lower than in the case of FCFS dispatching, with the exception of Manhattan, where the results from the two models are quite similar. This is understandable, given that inefficiencies in fragmented markets come from the presence of a stand-by fleet, represented by the *B* constant term in Eq. (2) that is necessary to ensure that an operator can respond to *any* trip request in its service area within a reasonable time. On the other hand, if perfect knowledge of upcoming demand is available, no such fleet is needed; idle vehicles can then be relocated in an ideal fashion, to start locations of upcoming trips. This is achieved in the ideal dispatching strategies generated by the Oracle model^10^. We show absolute fleet sizes as a function of demand for the oracle model in Fig. S8 of the Supplementary Information; we can observe that it is well approximated with a linear relationship in the form of Eq. (2). The value of the *B* constant is significantly lower than in the case of the FCFS model in all cities with the exception of Manhattan, where an almost zero *B* value was found in the FCFS model already.

However, *perfect* estimation of upcoming demand will not be likely achieved by any predictive model. Prediction errors are expected to depend on density according to the law of large numbers, putting operators with smaller demand shares still at a disadvantage. This way, especially for cities with a low total demand pool and fragmented markets, a larger stand-by fleet will likely still be needed to be able to guarantee *reliable* service, making the results of the FCFS model relevant even if better demand prediction models were to be implemented by operators.

## Discussion

While competition in on-demand mobility is expected to bring benefits in terms of lower prices and a better service quality to passengers, our results clearly show that, in the current non-cooperative model where demand is fragmented between the mobility operators, there are also significant potential costs in terms of overall fleet size increase. Not only our research confirms the importance of calculating inefficiencies due to demand segmentation, but it also outlines that these vary largely among cities and operators. Namely, increased demand segmentation will result in a much lower fleet size increase in cities with denser demand and higher traffic speed, while such effects can be especially significant if the original size of the demand pool is smaller or the traffic speed is lower. E.g. in Manhattan, we estimate that each additional operator adds only 2.5% to the total number of vehicles; in San Francisco, this number is 37%, and in Curitiba it is 67%. With the recent disruptive entry of TNCs into the urban mobility market, we expect that inefficiencies due to demand segmentation are already contributing to increasing congestion and emissions in cities.

Inefficiencies are unevenly distributed among operators, with smaller operators affected to a much larger degree. Since these inefficiencies directly translate to lower earnings and higher costs, smaller platforms will have a significant disadvantage in competing against larger players. This constitutes in essence a “rich-get-richer” effect, where larger players can rely on their efficiencies of scale to outcompete smaller ones. A newly created operator with limited resources will have a short time window to establish itself and gain a suitably large share of the demand to achieve financial sustainability. This observation is consistent with the fact that in many cities, typically one operator achieved near-monopoly, after intermittent periods of intensive competition from challengers. Our results can help establish such thresholds that operators need to achieve to be able to continuously compete.

It is important to observe that the above discussion applies to the current model where competing operators draw upon segmented demand and do not cooperate with each other in serving the overall demand pool in the city. As explained in the introduction, the inefficiencies in fleet management outlined in this paper are induced by a segmentation of demand, not necessarily of the market. In principle, it is possible to conceive a new model in which competing operators draw upon a *shared* demand pool, thus lessening the fleet inefficiencies outlined in this paper. A possible way of transitioning towards a shared demand pool model would be through policy/regulations. For instance, the city, or a third party authority, might be the only collector of demand for on-demand mobility from passengers via an open API that any third-party mobile app and mobility operator can connect to. Operators, including both taxis and TNCs would then compete to better serve a share of the overall demand pool available on this platform.

Most efficient operations could be achieved by a centralized (or coordinated) assigment of passengers to vehicles from *any* on-demand mobility operator in a city. On the other hand, competition between operators could be facilitated if passengers can choose an operator for each trip based on instantaneous offers including waiting times and price. This would provide the benefits of market segmentation while limiting the drawbacks coming from demand segmentation. Compared to the current situation where passengers need to open separate apps with instantaneously changing prices, such a system would move the burden of effective competition away from the end user, similarly to how price comparison platforms have done in the airline industry.

Such a platform would also facilitate sharing knowledge of the overall mobility demand in the city amongst all mobility operators, allowing them to develop advanced demand prediction tools that are key to efficient and pro-active vehicle relocation. Having access to improved demand knowledge would also allow a small operator entering the market to develop very accurate vehicle relocation strategies. Currently, smaller operators have access only to very limited demand knowledge (the small portion of demand currently served by their service), hampering their ability to develop accurate demand estimation tools and thus being in an even more disadvantaged position when competing with the larger operators in the market.

While our results clearly outline the benefits of a shared platform for on-demand urban mobility, they also provide guidance for cities in the current situation. Using the model for calculating the cost of demand segmentation proposed in our paper, policymakers could make more informed decisions on the number of operators allowed in a city, fleet-wide utilization requirements and congestion surcharges for each operator based on their demand share. In accordance with Eq. (6), the city-wide increase in fleet size only depends on the total number of operators; on the other hand, in accordance with Eq. (3), operators with different market shares are expected to have different fleet-wide utilization. Based on this, cities will want to limit the total number of players allowed in a market, while creating a framework that favours smaller operators, helping to ensure long-term competition. We emphasize that proactive action from regulators will be necessary, as currently TNC operators are not incentivized to limit the number of drivers on their platforms, thus we expect that fleet sizes are even higher than the minimum numbers considered in our work.

Finally, we note that our results point to some important future research directions. The operation model outlined above with a shared platform that allows passengers to directly combine mobility offers will require new strategies with regards to both pricing and empty vehicle relocation and raises important questions about the inherent trade-offs that arise, e.g. between price and waiting time. Furthermore, the connection between increased competition and total travel volume and related policy tools should also be considered. To facilitate future research in this domain, we share our simulation code and detailed examples on how to run it on the openly available Manhattan city taxi dataset as an open-source software^27^.

## Methods

### Dataset

The whole taxi dataset for Singapore includes close to 31 million taxi trips for the length of 77 days in 2012. The dataset for Manhattan includes almost 150 million taxi trips made in 2011 and is publicly available at^28^, while the one for San Francisco includes close to 800,000 trips from a 3 week period in 2008. The Datase for Vienna consists of almost 300,000 trips from a 1 month period in 2011. Finally, the Curitiba dataset covers the whole year of 2015 with 5.7 million trips in total. We note that the datasets include only trips that were successfully served, and do not include information about *lost demand*, i.e. trips that did not happen due to the unavailability of drivers.

In Manhattan, the dataset includes all trips served by the Yellow Cabs, and thus can be considered to include all demand (while excluding trips with destinations outside Manhattan), while in the other cities, the data includes trips served by only one operator with a varying market share. Nevertheless, we consider the trips present in the taxi dataset as the overall demand pool for each city in our main analysis. Having access to data from different locations and of different density allows us to test and verify our model for demand segmentation under a wide range of scenarios. On the other hand, our results can be easily adapted for different demand estimates as is demonstrated by the synthetic datasets generated based on real taxi data with the methods described in the SI.

In our analysis we focused only on workdays (i.e. Monday to Friday) as in our previous papers (e.g.^29–31^) we have shown that mobility patterns can significantly vary between workdays and weekends. Moreover, in order to minimize the effects of different dataset lengths, measured in the number of days that the data was collected on, from each dataset we chose only 3 typical weeks (i.e. 15 days). This limitation came from the San Francisco dataset having the smallest number of days recorded across all cities. For more information on how a typical week was chosen for each case, please refer to the SI.

### Simulations

In order to quantify the cost of demand segmentation, we first sampled a certain demand-share ratio from the original dataset. In this way, by sub-sampling the original demand, we simulated an operator with a certain market share. Then for each demand segment, we run an algorithm to calculate the fleet needed to serve the given demand. In other words, we assume that each passenger is using only one TNC application and can be assigned to a vehicle of only one operator. By repeating our estimation for samples of 10–100% of the full set of trips, we simulate market operators holding 10–100% of demand share. Finally, the measure we use to estimate the cost of demand segmentation is calculated as the percentage of additional TNC vehicles needed in the city to serve the given demand when the market is segmented compared to the case when the entire demand pool is served cooperatively by a single, centrally coordinated fleet.

Generally speaking, our approach consists of the following steps: (1) Define a strategy for dispatching vehicles to requests; (2) Define a strategy for relocation/cruising of idle vehicles; (3) Given a number of trips $N_T$, find the minimum fleet size $N_V$ that provides adequate service under the conditions above.

#### Dispatching

In this work, the main approach we use for simulating taxi dispatching is a First-Come-First-Served (FCFS) model, where each request is always assigned the closest available (idle) vehicle. If no suitable vehicle is found (either because there are no idle drivers or the waiting time would be too long), the request is rejected. This essentially assumes that all trips are booked via a central system (dispatch or an app) and does not consider the possibility for a street hailing. This matches well with the operational model of TNCs, while we note that street hailing can be considered a special case of the above booking process when the closest vehicle is immediately available. In practice, to consider a certain fleet size adequate, we require at least $z = 95\%$ of trips to be successfully served within $5\,\mathrm {min}$ waiting time. Allowing some of the trips to be unserved accounts for the presence of an unknown lost demand in our datasets, and it is also necessary since without advance knowledge of trip requests, ensuring all trips to be served would require unrealistically large fleet sizes.

#### Relocation

We employ a simple random cruising strategy for relocating idle drivers. Whenever finishing a trip, a vehicle selects a random “target node” (from the set of discrete nodes considered as the simulation space) and moves there using the fastest possible route. Upon reaching the target node, the vehicle stays there, idling. Vehicles can be assigned to new trips during both idling or while on the move to their selected target node. Target nodes are selected with probabilities proportional to the number of trips starting there during a training period; this emulates that drivers are expected to have a knowledge about trip demand patterns in general. This is a simplified model for vehicle reallocation as: (1) time of day is not considered when making a choice for target nodes; (2) vehicles select target nodes independently at random, there is no coordination among them; and (3) travel distance is not taken into consideration when selecting a target node. Nevertheless, as the goal of this paper is to compare demand segmentation effect across different cities, even this simplified model serves its purpose as the same limitations are observed in each city.

#### Finding minimal fleet size

Given the above simulation methodology, we then consider demand as a set of trips of size $N_T$, either from the real dataset, a sample of trips chosen from it, or generated via a random process. We perform repeated simulations varying $N_V$ until we arrive at a minimal fleet size that is sufficient to serve $z N_T$ trips. In practice, we use a binary search, as described by Algorithm S1 in the SI. This approach is fundamentally different from the optimization-based methods from related work^10,13,14^, but allows us to obtain the results that are more readily and generally applicable to real-world taxi and TNC operations, since they do not depend on having an advance knowledge of demand.

## Supplementary Information

Supplementary Information.
