# Supplementary material for: The cost of non-coordination in urban on-demand mobility
Source: Sci Rep. 2022 Mar 18;12:4669. doi: 10.1038/s41598-022-08427-2 (PMC8933415; doi:10.1038/s41598-022-08427-2)
Supplement: Supplementary file 1 — Supplementary Information. [file 41598_2022_8427_MOESM1_ESM.pdf]

The cost of non-coordination in urban on-demand mobility

Dániel Kondor, Iva Bojic, Giovanni Resta, Fábio Duarte, Paolo Santi, Carlo Ratti

Supplementary Information

Datasets

In addition to the taxi datasets, we also downloaded the road networks for our five cities from OpenStreetMap[1]. For Singapore we used a bounding box that covers the whole island, for New York the one that includes only Manhattan, and for San Francisco, Vienna and Curitiba we determined bounding boxes based on where the majority of taxi trips happened. After downloading initial road networks, for Singapore we manually excluded roads that provide connections to Malaysia, and finally, kept the largest connected component of the resulting road network. We further processed the raw networks by performing a friend-of-friend clustering, grouping together nodes with a threshold radius of 20 m, reducing the network size to simplify processing and remove uncertainties from small errors in Global Positioning System (GPS) data. After the clustering procedure, the road network for Singapore had almost 12,000 nodes, the one for Manhattan had more than 4,000 nodes and the networks for San Francisco, Vienna and Curitiba had around 10,000, 16,000 and 20,000 nodes, respectively. Trip start and end locations were matched to the closest node in these road networks, and the simulation presented in the next section then considered space on the granularity of network nodes. Travel times between each pair of the nodes were estimated on a hour-by-hour basis using the taxi dataset in each city with the algorithm from Ref.[2].

Some of the characteristics of different taxi datasets are shown in Table S1. We compared the area of each city expressed in km^2. The area of New York in this study is limited to the borough of Manhattan and thus is the smallest among all five cities, while the area of Singapore is the largest. Although covering the smallest area, the total number of recorded taxi trips in Manhattan is the highest, making it the case with the highest number of trips per day per km^2. The smallest density is recorded in Vienna, Austria. The longest trips of almost 10 km on average are recorded in Singapore, while in Curitiba trips are on average 3 times shorter. Even though the trips in Curitiba might be the shortest in length, they last the longest in time, making the average traffic speed in Curitiba the lowest one of only 7 km/h. For comparison, the average traffic speed in Vienna is almost 45 km/h, which is more than 6 times faster.

	**Singapore**	**Manhattan**	**San Francisco**	**Vienna**	**Curitiba**
**City size (km^2)**	366	45	100	258	213
**Number of trips**	6,092,054	6,244,251	497,818	138,605	260,362
**Trips per day per km^2**	1110	9251	323	36	82
**Avg. peak hour trips per km^2**	65.95	620.5	22.99	2.319	9.049
**Avg. trip length (km)**	9.72	3.68	5.14	7.03	3.09
**Avg. trip duration (min)**	14.89	11.12	10.93	9.37	25.06
**Avg. trip speed (km/h)**	39.18	19.86	28.21	44.97	7.39

Table S1. General information about the characteristics of different datasets.

Calculating a typical week

For for each workday (i.e. Monday to Friday) in each city, we calculated the average number of trips. Then, we went through our data, and for each week we calculated the total difference between that week and the calculated average. We sorted weeks in a descending order and chose the first three weeks which we then call typical weeks.

Materials and methods

The methodology used in this paper presents a simple, but a realistic approximation of ride-hailing operations. The included cruising step ensures that the distribution of idle vehicles asymptotically follows the distribution of demand in the city. Note: although our simulation assumes that drivers are on the road for a whole day (i.e. a 24-hour period), it could be easily extended to consider shorter shifts, and changes of drivers at given times of day. Furthermore, since trip demand is typically not constant during the day, we expect that different times of day could require different effective fleet sizes. A further extension of our work could consider "covering" the day with fixed-length (e.g. 8 hour) driver shifts in an optimal way to serve at least N_{min} trips. We consider this analysis however to be beyond the scope of the current work.

An alternative approach, which could give a more concrete understanding of specific fleet needs in a city, could aim at finding the fleet size that gives a sufficient performance given a specific sequence of trips. This way, any real data can be thought of as training data instead of test data. Nevertheless, if the strategy is generic (i.e. it is not influenced by daily variation

of demand and is trained on a sufficiently large set of days), the result can still provide a good understanding of the possibility to serve trips with a given efficiency. Assuming this approach, after defining the strategies for fleet management, a binary search was used to find the minimum fleet size that provided an adequate level of service given a concrete set of trips. The whole procedure of finding the minimum fleet size to serve the given demand is outlined in Algorithm 1.

Algorithm 1 Basic binary search algorithm to find the minimum fleet given a dispatching strategy, a set of trips and target measures z and T_{wait}.

S: fleet management strategy (including dispatching and cruising)
T: set of trips in a day
N: initial guess of fleet size
Δ: initial step size for varying the fleet size
$N_{\text{min}} = z|\text{T}|$: target number of trips to serve
T_{wait}: maximum acceptable waiting time
$D =$ None: direction of last step taken
while True **do**
    Simulate serving trips in T according to strategy S with N vehicles
    set N_1 to the outcome, i.e. the number of trips successfully served within T_{wait}
    if $N_1 = N_{\text{min}}$ **then**
        break
    end if
    if $N_1 > N_{\text{min}}$ **then**
        if $\Delta = 1$ and $D =$ Up **then**
            break ▷ avoid an infinite loop if the exact number of target trips cannot be reached
        end if
        $D =$ Down
        $\Delta = \max(\Delta/2, 1)$
        $N = N - \Delta$
    else
        $D =$ Up
        $\Delta = \max(\Delta/2, 1)$
        $N = N + \Delta$
    end if
end while
Result: N, the minimum fleet size required to serve at least N_{min} trips

Calculating fleet size factor R(p)

Since trip requests arrive in an arbitrary manner, at any time, we need to have a set of available drivers ready to be assigned to any new request. Specifically, if we would like to ensure that trip requests can be served within T_{wait} maximum waiting time with a high probability, then either of the following needs to be true: (1) there should be trips already happening that will finish within T_{wait} and in a location that allows the driver to reach the new passenger in a short time; or (2) there should be idle drivers distributed in the city in a way that the new passenger's location can be reached by at least one driver within T_{wait}. The first possibility primarily depends on the origin-destination distribution of the trips; operators' dispatching choices will only influence to what extent any optimisation opportunities are exploited, but cannot result in more efficient operations than what is allowed by the structure of trip demand in the city. The second possibility essentially corresponds to deploying a "standby fleet" to cover areas of the city where imbalances in trip origins and destinations would result in requests being unserved. The size of this is primarily determined by the geometry of the city, the traffic speed, and the structure, but not the density of demand, thus we represent this by the constant term B. In general, we expect B to scale linearly with city area, i.e. $B = b|\mathscr{C}|$ for city $\mathscr{C}$.

Given the relationship between the number of trips and the fleet size from Eq. (2) from the main text, we can derive a simple analytical expression for the fleet size factor described by Eq. (1):

$$R(p) = \frac{b|\mathscr{C}|}{AN_T + b|\mathscr{C}|}\left(\frac{1}{p} - 1\right) = \frac{b}{An_T + b}\left(\frac{1}{p} - 1\right) \quad (1)$$

where n_T is the average trip density, i.e. $N_T = n_T|\mathscr{C}|$. By dividing everything by the city size $|\mathscr{C}|$, in the end we use b as the size-independent scaled version of the constant B from Eq. (2).

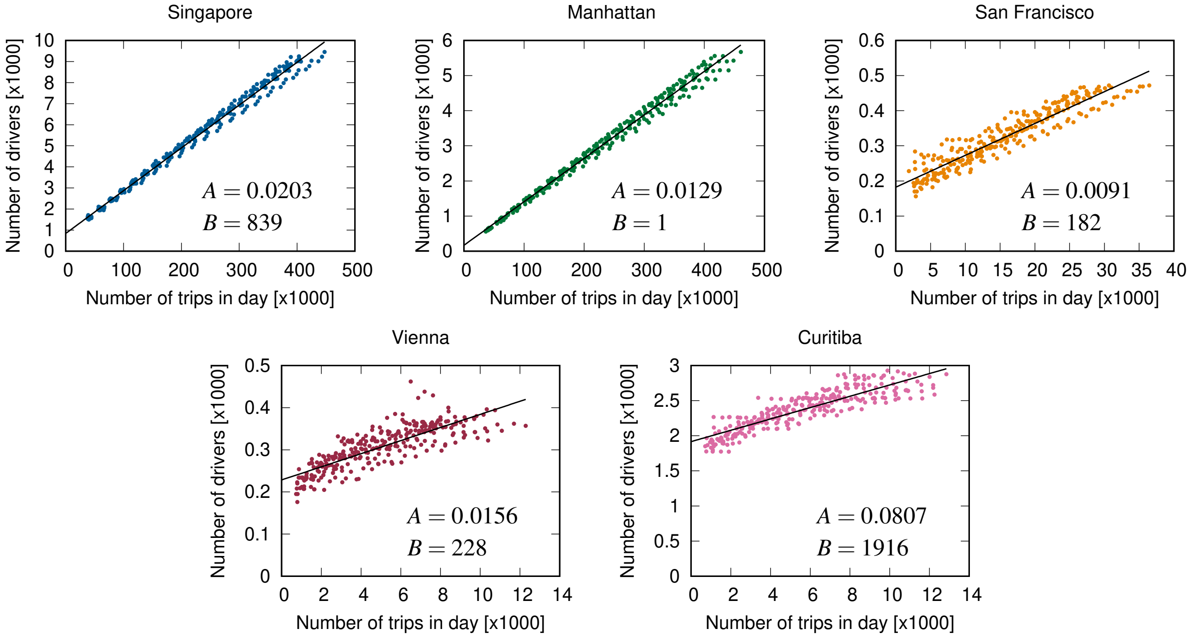

Figure S1. Verifying the linear relationship between the daily number of trips and the number of TNC vehicles required. Number of drivers is estimated as $N_V = AN_T + B$, where the best-fit A and B parameters for each city are shown in the panels.

Generating trips based on a random model

Beside the real trips, we further use the model presented in[3,4] to generate trips in a random process. This allows us to generate trips with different presumed densities, essentially allowing us to ask the question "What if we start from a larger number of initial trips?" or "What if there is a larger demand pool originally?". This also allows us to test more thoroughly how the factors in the previous model depend on the density of trips.

For each city, we generated 9 synthetic datasets, using the distribution of trips inferred from the real data; for each synthetic dataset, we generated 15 days of trips, similarly to the real data. This means that for each city, we had a total of 10 datasets (one original and 9 synthetic). For each dataset, we confirmed that fleet size factors can be modeled in the form presented as Eq. (3) in the main text, and calculated the best-fit D parameter. In Figs. S2, S3 and S4, we display these D values as a function of average trip distance, average trip duration, and average traffic speed respectively. Points are grouped by color based on trip density, while points with the same x-axis value correspond to simulations carried out in the same city (as we have 5 cities in the dataset, each of the figures has five distinct values on the x-axis).

Effect of peak daily trip rate

Our main analysis considers the number of trips in a day as the main starting point for modeling fleet size requirements and the effect of market segmentation. While this presents a simple and straightforward measure that can be easily applied to any city, an additional complexity comes from the fact that the distribution of demand in a day is not uniform. This means that the required fleet size will likely be affected by the *peak* demand in a day. To assess the importance of this, we calculated a measure of "peak demand" for each day in our datasets as the hourly maximum number of trips, and a measure of "peak utilization" as the maximum of hourly total trip duration. We display the estimated fleet size as a function of these variables in Figs. S6 and S7. We see that this dependence can be well approximated by a linear relationship as well, with significant variation among the coefficients among the cities.

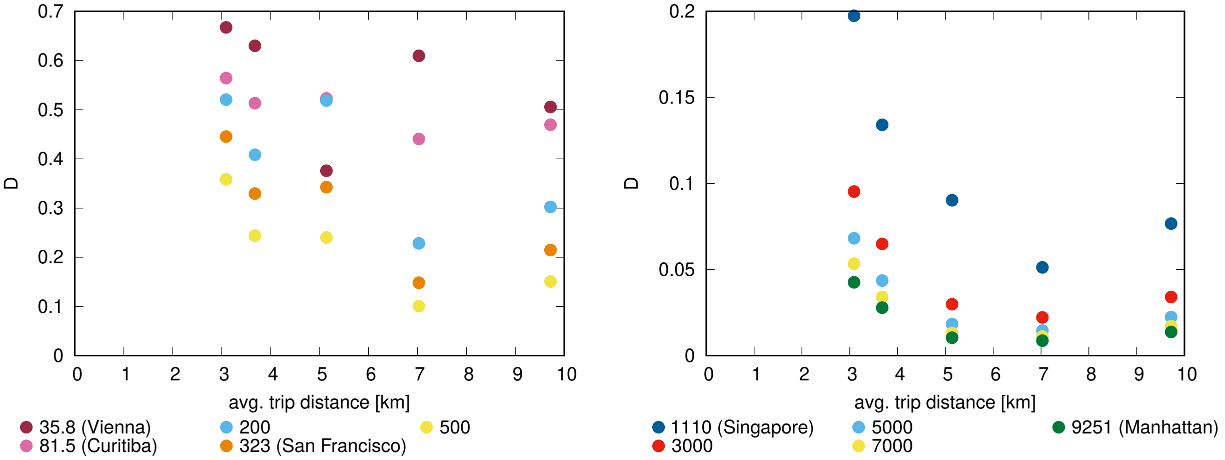

Figure S2. Constant D as a function of the average trip distance in the synthetic datasets. Each set of points belongs to a set of results with a given density, displayed in the legend as trips per day per km^2. What we can observe is that there is no direct correlation between the average trip distance and the constant D.

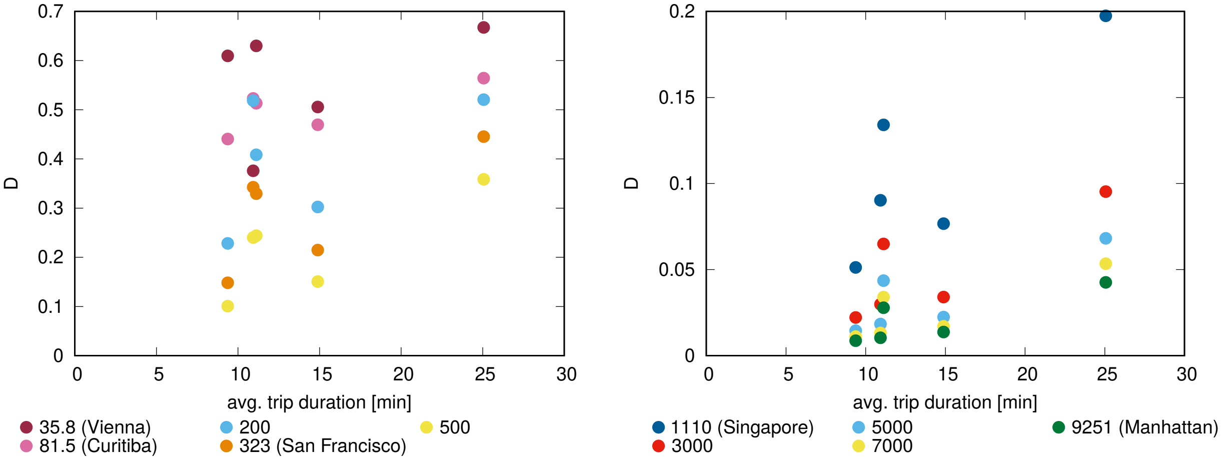

Figure S3. Constant D as a function of the average trip duration in the synthetic datasets. Each set of points belongs to a set of results with a given density, displayed in the legend as trips per day per km^2. What we can observe is that there is no direct correlation between the average trip duration and the constant D.

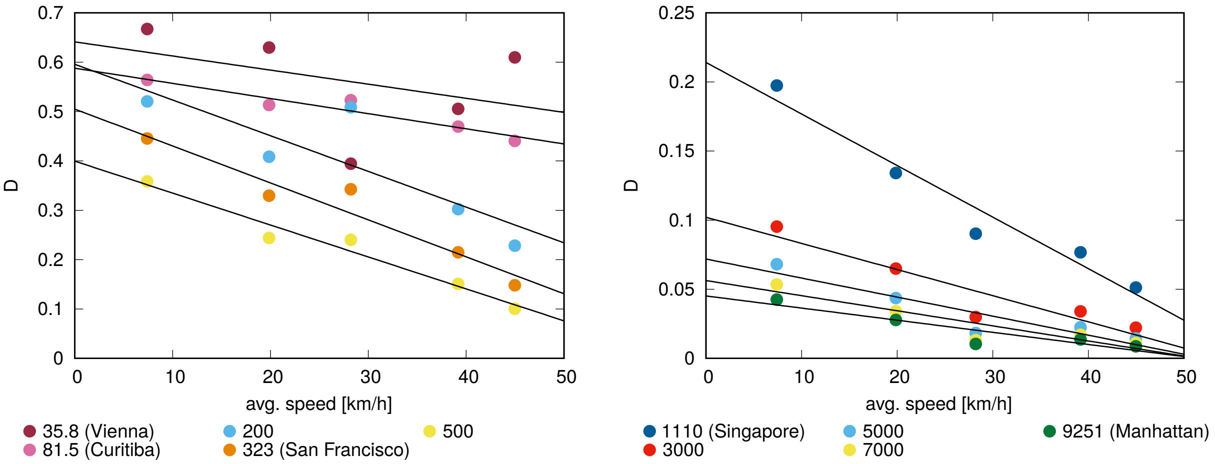

Figure S4. Constant D as a function of the average travel speed in the synthetic datasets. Each set of points belongs to a set of results with a given density, displayed in the legend as trips per day per km^2. What we can observe is that the constant D decreases monotonically with an increase of the traffic speed.

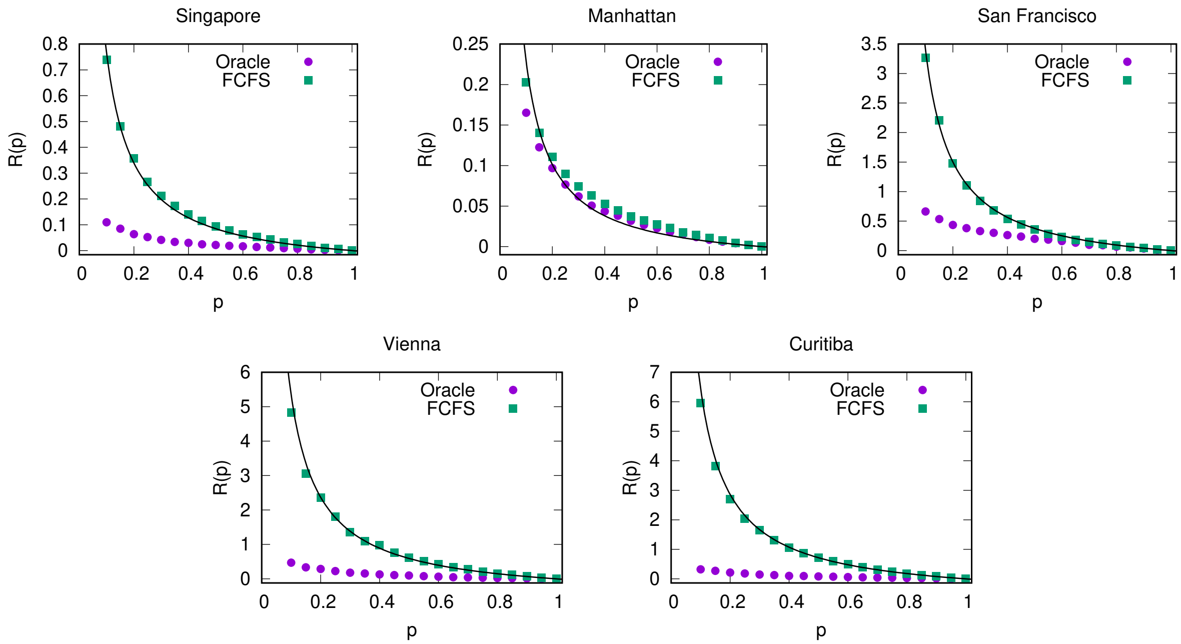

Figure S5. Results of calculating fleet size factors in the Oracle model, compared to the FCFS results. As expected, when an advance knowledge of upcoming demand is available, as used in the Oracle model, then the cost of non-coordination is lower.

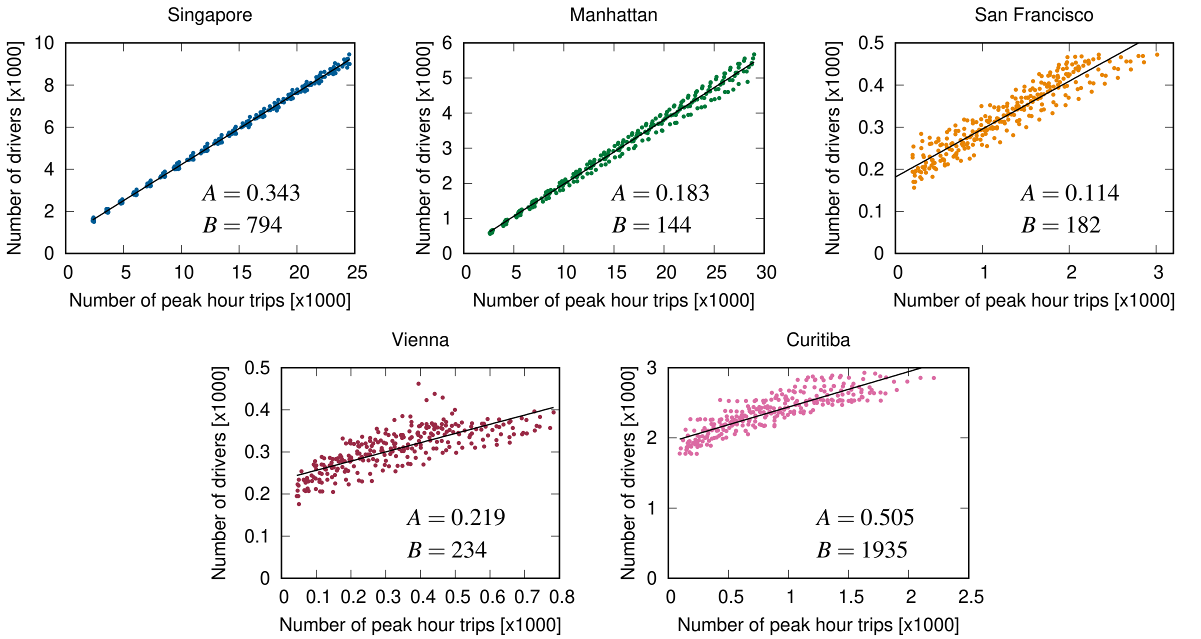

Figure S6. Number of vehicles in the fleet as a function of peak trip rate (i.e. the number of trips in the peak hour of each day in the dataset).

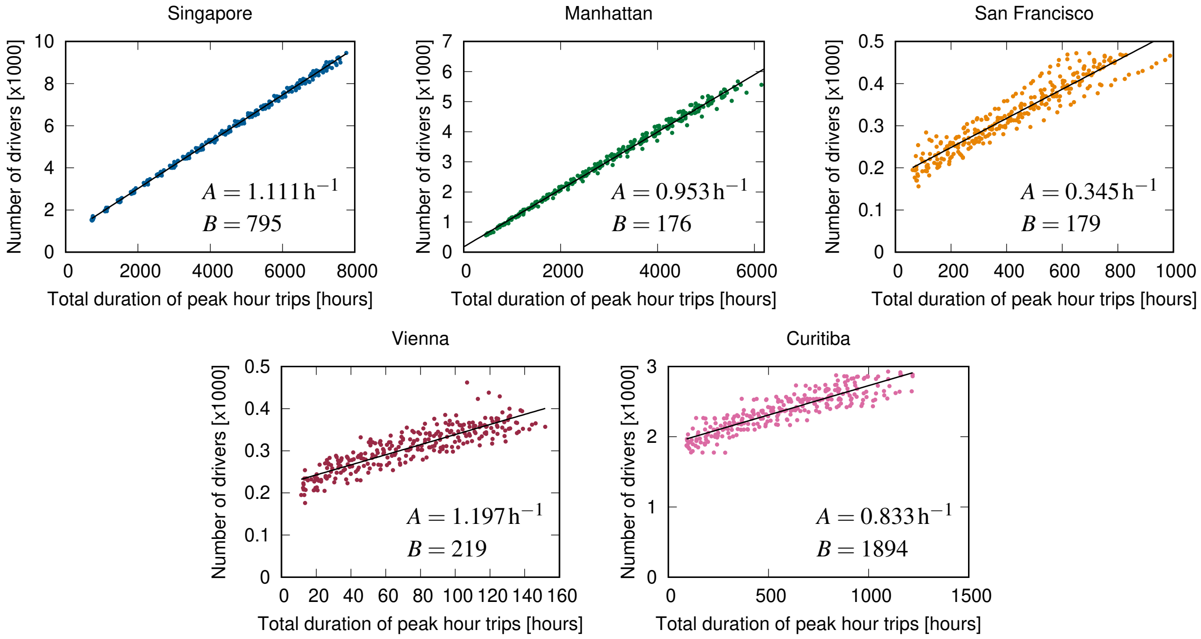

Figure S7. Number of vehicles in the fleet as a function of peak trip duration (i.e. the total duration of trips in the peak hour).

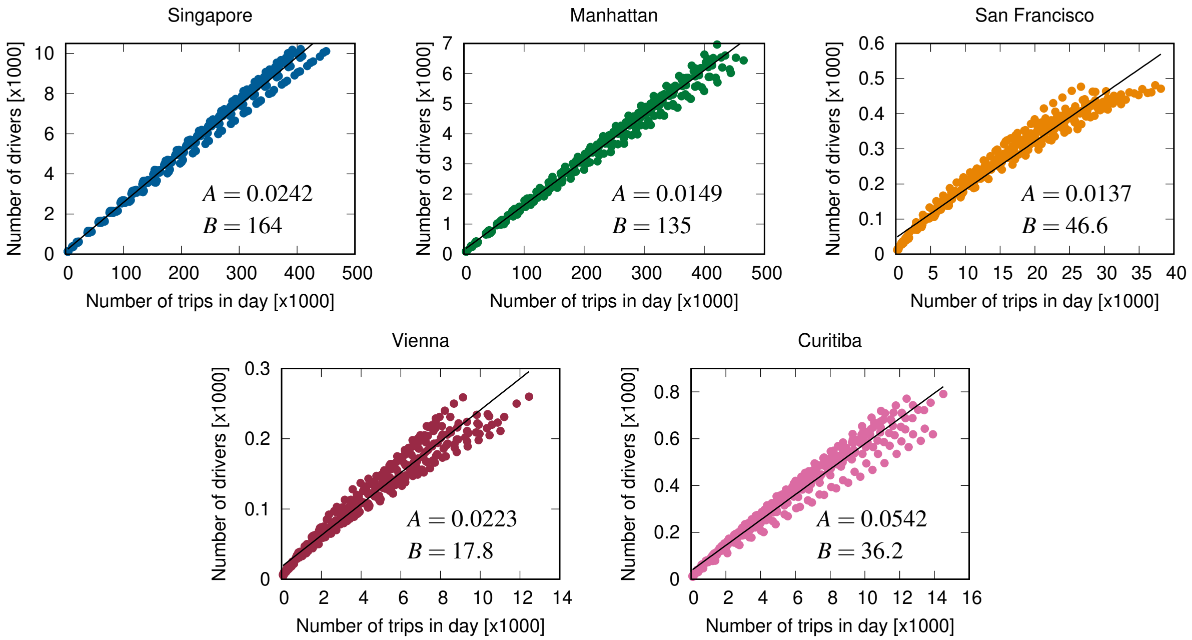

Figure S8. Linear fit of fleet size as a function of daily trip numbers in the Oracle model. Note that the fleet sizes displayed in this figure are those required for serving all trips without any delay and thus can be larger than fleet sizes in the FCFS model which are only required to serve 95% of trips with a maximum delay of 5 min. Extension of the oracle model to select an "ideal" subset of trips or to allow flexible delays for trip start times leads to a prohibitive increase in combinatorial complexity of the problem[5].

References

1. Haklay, M. & Weber, P. Openstreetmap: User-generated street maps. *IEEE Pervasive Comput.* **7**, 12–18 (2008).
2. Santi, P. *et al.* Quantifying the benefits of vehicle pooling with shareability networks. *PNAS* **111**, 13290–13294, DOI: 10.1073/pnas.1403657111 (2014).
3. Sagarra, O., Szell, M., Santi, P., Díaz-Guilera, A. & Ratti, C. Supersampling and network reconstruction of urban mobility. *PLoS One* **10**, e0134508 (2015).
4. Tachet, R. *et al.* Scaling Law of Urban Ride Sharing. *Sci. Reports* **7**, 42868, DOI: 10.1038/srep42868 (2017).
5. Bertsimas, D., Jaillet, P. & Martin, S. Online vehicle routing: The edge of optimization in large-scale applications. *Oper. Res.* **67**, 143–162, DOI: 10.1287/opre.2018.1763 (2019).
